# Can variants, reinfection, symptoms and test types affect COVID-19 diagnostic performance? A large-scale retrospective study of AG-RDTs during circulation of Delta and Omicron variants, Czechia, December 2021 to February 2022

**DOI:** 10.2807/1560-7917.ES.2023.28.38.2200938

**Published:** 2023-09-21

**Authors:** Tomáš Kliegr, Jiří Jarkovský, Helena Jiřincová, Jaroslav Kuchař, Tomáš Karel, David Chudán, Stanislav Vojíř, Michal Zavřel, Ondřej Šanca, Ruth Tachezy

**Affiliations:** 1 Department of Information and Knowledge Engineering, Faculty of Informatics and Statistics, Prague University of Economics and Business, Prague, Czechia; 2 Institute of Health Information and Statistics of the Czech Republic, Prague, Czechia; 3 Institute of Biostatistics and Analyses, Faculty of Medicine, Masaryk University, Brno, Czechia; 4 National Reference Laboratory for Influenza and Respiratory Viruses, National Institute of Public Health, Prague, Czechia; 5 Department of Software Engineering, Faculty of Information Technology, Czech Technical University, Prague, Czechia; 6 Department of Statistics and Probability, Faculty of Informatics and Statistics, Prague University of Economics and Business, Prague, Czechia; 7 Department of Genetics and Microbiology, Faculty of Science-BIOCEV, Charles University, Prague, Czechia; * These authors contributed equally to this article and share the first authorship

**Keywords:** reinfection, vaccination, SARS-CoV-2, rapid antigen test, omicron, delta

## Abstract

**Background:**

The sensitivity and specificity of selected antigen detection rapid diagnostic tests (AG-RDTs) for SARS-CoV-2 were determined in the unvaccinated population when the Delta variant was circulating. Viral loads, dynamics, symptoms and tissue tropism differ between Omicron and Delta.

**Aim:**

We aimed to compare AG-RDT sensitivity and specificity in selected subgroups during Omicron vs Delta circulation.

**Methods:**

We retrospectively paired AG-RDT results with PCRs registered in Czechia’s Information System for Infectious Diseases from 1 to 25 December 2021 (Delta, n = 20,121) and 20 January to 24 February 2022 (Omicron, n = 47,104).

**Results:**

When confirmatory PCR was conducted on the same day as AG-RDT as a proxy for antigen testing close to peak viral load, the average sensitivity for Delta was 80.4% and for Omicron 81.4% (p < 0.05). Sensitivity in vaccinated individuals was lower for Omicron (OR = 0.94; 95% confidence interval (CI): 0.87–1.03), particularly in reinfections (OR = 0.83; 95% CI: 0.75–0.92). Saliva AG-RDT sensitivity was below average for both Delta (74.4%) and Omicron (78.4%). Tests on the European Union Category A list had higher sensitivity than tests in Category B. The highest sensitivity for Omicron (88.5%) was recorded for patients with loss of smell or taste, however, these symptoms were almost 10-fold less common than for Delta. The sensitivity of AG-RDTs performed on initially asymptomatic individuals done 1, 2 or 3 days before a positive PCR test was consistently lower for Omicron compared with Delta.

**Conclusion:**

Sensitivity for Omicron was lower in subgroups that may become more common if SARS-CoV-2 becomes an endemic virus.

**Key public health message**

**What did you want to address in this study?**

The performance of SARS-CoV-2 antigen tests can be influenced by the circulating virus variant but also by changes in the characteristics of the population which is building immunity through vaccination and reinfection. We wanted to compare the performance of antigen tests for the Delta versus the Omicron variant.

**What have we learnt from this study?**

For the Omicron virus variant, there is a small decrease in the ability of rapid tests to identify individuals who were both infected and vaccinated. There are indications of a possibly larger decrease when the rapid tests are conducted outside the period of the highest viral load, i.e. shortly after infection. Rapid antigen tests continue to be the most reliable when the tested person is symptomatic.

**What are the implications of your findings for public health?**

PCR tests should be preferred for SARS-CoV-2 testing, especially in asymptomatic individuals. Antigen tests for Omicron may be more likely to give false negative results in the first few days after infection than for Delta. On the other hand, antigen tests are useful for the diagnosis of symptomatic disease. It is advisable to choose antigen tests that are on the European Union Category A list.

## Introduction

Rapid antigen tests play an important role in the public response to the spread of severe acute respiratory syndrome coronavirus 2 (SARS-CoV-2). Their effective use depends in large part on accurate and detailed information on their diagnostic performance as this may evolve over time. By May 2023, several Omicron sublineages were in circulation, many infected individuals had been vaccinated and a large share of infections were reinfections. While prior research has largely concentrated on the effect of vaccination and previous infection on the risk of SARS-CoV-2 infection (e.g. [1,2]), the first objective of our study was to address the paucity of research on the effect of these factors on the diagnostic performance of AG-RDTs.

According to prior research, the success of antigen testing partly depends on the quality of the tests used. It has been shown that multiple AG-RDTs used in Czechia did not meet the World Health Organization (WHO) criteria for sensitivity [3]. A comprehensive review has found that issues with lower sensitivity of antigen tests are common, which raises the question of whether antigen tests are suitable for screening programmes [4]. Some international bodies such as the WHO and the European Union (EU) have compiled evidence from multiple sources and released lists of approved antigen tests to facilitate their selection. Once a test has been put on a list, its performance is subject to further surveillance, with a possibility of its withdrawal from the list at a later date. This process may, however, be slow to react to changes in the virus or in the underlying population characteristics and variations in AG-RDT quality. The second objective of our study was to evaluate the main AG-RDT list used in Europe, the EU Common List [5], evaluate its recent split into Category A and Category B, and compare it with the WHO Emergency Use Listing (EUL).

**Figure 1:**
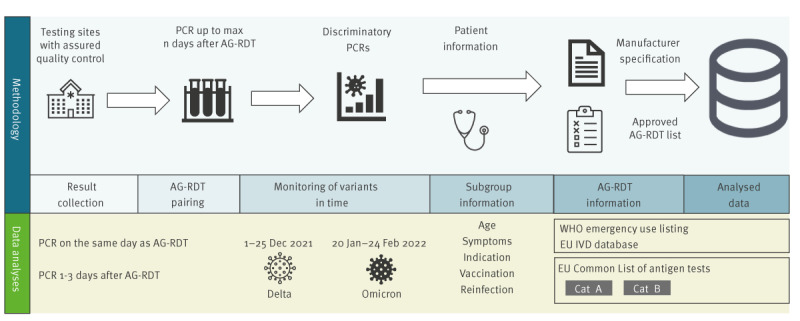
Methodology and enrichment data sources, retrospective study of AG-RDT performance during circulation of Delta and Omicron variants, Czechia, December 2021–February 2022

AG-RDT: antigen detection rapid diagnostic test; Cat: category; EU: European Union; IVD: in vitro diagnostic medical devices; WHO: World Health Organization.

To address these research questions, we present a new methodology based on analysing AG-RDTs and PCRs that have been registered as part of the state-mandated logs of AG-RDT and PCR results, which have been further enriched with additional data (Figure 1). We demonstrate the methodology by analysing nearly 70,000 pairs of AG-RDT and PCR samples, comparing multiple subpopulations and test characteristics.

## Methods

Data for this research were collected in Czechia (population 10.5 million) from December 2021 to February 2022.

### Collection of SARS-CoV-2 testing results

The analysed data originated from the official Czech Information System of Infectious Diseases (ISIN) database, which records AG-RDT and PCR tests performed in hospitals, by general practitioners and at official testing sites as mandated by the applicable legislation. AG-RDT and PCR testing were often conducted at the beginning of the infection as Czechia did not require a confirmatory test to end the isolation or quarantine period.

### The pairing of AG-RDT and PCR results

Out of all collected results, we first selected only those where the same person had taken AG-RDT and PCR on the same day. Note that as justified in prior work [6], taking both tests on the same day may be indicative of a higher viral load given how testing in Czechia was organised. In the study period, there were longer waiting times for PCR tests than for AG-RDTs, which could be done at a wider network of testing sites, including general practitioners. Furthermore, there was a quota for the maximum number of PCRs and AG-RDTs per person that were covered by insurance [7]; therefore, taking both tests at the same time would be more indicative of a suspected infection.

To evaluate the performance of AG-RDTs for the detection of an infection in a presymptomatic phase, we included three additional datasets. For these, we used those test pairs where the AG-RDT was performed on asymptomatic individuals 1 day, 2 days and 3 days before a PCR test and where the same person was symptomatic as reported for the PCR test.

### Monitoring of variants

For the purpose of the comparative analysis, we used the collected data to generate two datasets, one representing the SARS-CoV-2 Delta variant and one representing the SARS-CoV-2 Omicron variant. The Delta dataset consisted of confirmed SARS-CoV-2 cases recorded between 1 and 25 December 2021. During this period, the prevalence of Omicron in Czechia was very low. Until 17 December, only six cases of Omicron (BA.1) were detected with discriminatory PCR tests, which is 0.6% of 984 discriminatory PCR tests performed in the monthly reporting period covering 17 November to 17 December 2021 [8]. The remaining 99.4% of cases were infections with Delta subvariants [8]. 

Up until 25 December 2021, there were less than 10 daily Omicron cases based on a discriminatory PCR test (considering those who took an AG-RDT and within 3 days a subsequent positive PCR test; data not shown), therefore this date was chosen as the cut- off for the Delta dataset. After 25 December, the share of Omicron cases started to rise quickly. The Omicron dataset consisted of confirmed SARS-CoV-2 cases recorded in the period between 20 January and 24 February 2022. 

On 20 January 2022, the percentage of Omicron cases among SARS-CoV-2 cases was already at 96.2% (based on 4,011 discriminatory PCR tests [9]), further increasing to over 99.5% of cases recorded between 6 and 14 February (based on 49,879 discriminatory PCR tests done in 57 laboratories across Czechia [10]). An analysis of variants covering the period of 14 January to 14 February showed that the BA.1 Omicron variant was responsible for 52.9% of SARS-CoV-2 cases, BA.1.1 for 35.6% and BA.2 for 4.0%. The remaining share was split among Delta subvariants (5.2%) and other variants (2.3%) [10].

### Subgroup information

Each sample was associated with the same set of indicators that were used for subgroup analyses in [6], where these indicators are described in detail: reasons for the test (indication), vaccination status, age group, region-level incidence and presence of at least one symptom. 

The preventive reason was used mainly for tests of asymptomatic individuals at workplaces and for screening tests, the diagnostic reason for symptomatic patients and the epidemiological reason for contact tracing (refer to [6] for details). As in [6], a person was considered as vaccinated when at least 2 weeks after the first dose of the Janssen Ad26.COV2-S COVID-19 vaccine or 2 weeks after a second dose of any of the other vaccines approved in Czechia or vaccines approved abroad and recognised in Czechia. The age groups were 0–12 years, 13–18, 19–25, 26–59 and ≥ 60 years. The region-level incidence values were sourced from the official statistics [11].

In this analysis, we extended the set of indicators by the presence of specific symptoms: cough, muscle pain/joint pain/chills (feeling feverish), diarrhoea/vomiting, temperature, loss of smell/taste and other symptoms. The second new indicator was a previous SARS-CoV-2 infection, which was defined according to the European Centre for Disease Prevention and Control [12]: An AG-RDT sample was considered from someone with a previous SARS-CoV-2 infection if the same person had earlier tested positive with PCR or AG-RDT for SARS-CoV-2 and the AG-RDT was performed more than 60 days after the first positive result.

### AG-RDT information

We used the EU database of COVID-19 in vitro diagnostic devices (JRC DB) to retrieve metadata on SARS-CoV-2 AG-RDTs (downloaded in XML format on 30 August 2022). To do this, we performed automatic mapping of antigen detection rapid diagnostic tests (AG-RDTs) based on a combination of test and manufacturer names between ISIN and the JRC DB records, which resulted in 540 distinct AG-RDTs in ISIN mapped to the JRC DB records. For 34 additional AG-RDTs, the lack of an automatic match was resolved by manual mapping. The most common reasons included a spelling error or small changes in the manufacturer name and swapped manufacturer and test name. The manual mapping was done by an external annotator and checked by one of the authors (TKl). 

We used the mapped JRC DB records to determine whether the AG-RDT was used exclusively with saliva or not. As saliva tests, we considered AG-RDT tests that were marked in the EU database for use on saliva and not marked for use with any other sample type. Fluorescence immunoassays (FIA) were defined as those with ‘FIA’ (case-sensitive) in the test name. We also analysed the stated target proteins of AG-RDTs using the JRC DB. More than 99.9% of AG-RDT results in both analysed subsets were performed by AG-RDTs that were stated in the JRC database as using the nucleocapsid target protein or a combination of a nucleocapsid target protein and the spike protein, or no target protein was stated. Fewer than 0.1% of AG-RDT results were done with AG-RDTs using spike protein only according to the JRC database.

### AG-RDT lists

Our prior work [6] evaluated the EU list of COVID-19 antigen tests released on 6 May 2022 that contained a list of independently validated AG-RDTs. When the EU in vitro diagnostic medical device (IVD) regulation came into force, this list was split into the EU Category A list which requires that an AG-RDT is successfully evaluated by at least one prospective clinical field study and the EU Category B list which applies a less strict criterion of a retrospective in vitro study; both categories of studies also need to meet additional criteria [5]. This resulted in a substantial change compared with the original version of the EU list that did not make this distinction, which was evaluated in [6]. To issue COVID-19 certificates, the EU strongly encourages the use of its Category A list [5]. We therefore decided to include the new sublists as subgroups for our analysis here. The JRC DB does not contain information on which sublist a given AG-RDT is placed on. We therefore used the EU Common List of approved antigen tests [5] and then matched the included test identification number. Finally, we used the WHO EUL list of approved antigen tests [13], which contained eight AG-RDTs of which three were in our data according to the match between both the test and manufacturer name.

### Statistical analysis

We used exact Clopper–Pearson confidence intervals for binomial distribution for hypotheses testing to confirm that the approximation by normal distribution z-test could not lead to inaccurate results in situations where the sensitivity is within extreme limits close to 1 [14]. It follows from the observational character of the study that proportions of individual AG-RDT types used in the two compared periods were not identical. To evaluate to what extent changes in the distribution of AG-RDT types influenced the overall sensitivity, we proceeded as follows. We first determined the sensitivity of individual AG-RDT types as *sens_i_^p^ = TP_i_^p^/(TP_i_^p^ + FN_i_^p^)*. The value *TP_i_^p^* denotes the number of true positives, i.e. results recorded for AG-RDT of type *i* in period *p,* where the AG-RDT result was positive and a PCR test done on the same day was also positive. *FN_i_^p^* denotes the number of false negatives, i.e. results recorded for AG-RDT of type *i* in period *p* where the AG-RDT result was negative and a PCR test done on the same day was positive. For each AG-RDT, we also determined its share of the total number of positive PCR tests in the given period: *share_i_^p^ = (TP_i_^p^ + FN_i_^p^)/∑^n^_i=1_(TP_i_^p^ + FN_i_^p^),* where n is the number of distinct AG-RDTs (missing AG-RDT name is also included as a distinct type). For example, for the Delta period (denoted as Δ), the average sensitivity is computed as *sens^Δ^*  *= ∑^n^_i=1_ sens^Δ^_i_  × share^Δ^_i_* . To evaluate the effect of varying shares of individual AG-RDTs in the two periods, we compared this average with the weighted average sensitivity of AG-RDTs computed from sensitivities determined in one period and the distribution (share) of the tests from the other period. For Delta, we compared *sens^Δ^* with the average computed from the Delta period sensitivities but the share of the tests from the Omicron period (denoted as *o*): *sens^Δ^_o_*_-distr_ *= ∑^n^_i=1_ sens^Δ^_i_ × share^o^_i_*. Analogically, we compared *sens^o^* with *sens^o^_Δ_*_-distr_. A statistical z-score test for two proportions was used with a significance level of 5%. The results were confirmed by the exact binomial test for two proportions.

### Logistic regression

To analyse factors contributing to a false negative result of AG-RDTs, we created a multivariable logistic regression model. The target variable was binary, indicating whether AG-RDT and PCR were both positive (coded as 1) or whether the AG-RDT was negative, and the PCR test was positive (coded as 0). As independent variables, we used age group (as defined above), indication (reason to test), incidence level, presence of individual symptoms, vaccination and reinfection status. Records were excluded when a value was missing for one or more of the included predictor variables. We included only same-day tests. The analysis was performed using the statsmodels Python package (version 0.13.5) [15] and checked against the results of R package stats (R Foundation, version 4.2.2).

## Results

A summary of the collected data is presented in Table 1. Since the tests were conducted in different (though consecutive) periods, we also report a population-wide SARS-CoV-2 incidence, as based on official statistics.

**Table 1:** Overview of data on SARS-CoV-2 antigen detection rapid diagnostic tests from the Information System of Infectious Diseases, Czechia, December 2021–February 2022 (n = 67,225)

	Delta				Omicron			
Data collection period (inclusive)	1–25 Dec 2021				20 Jan–24 Feb 2022			
Time window of data collecting (in days)	25				36			
Average 14-day incidence per 100,000^a^	1,862				3,813			
Exported from ISIN in	February 2022	March 2023			February 2022	March 2023		
Days between AG-RDT and PCR	Same day	1	2	3	Same day	1	2	3
Symptoms	No restrictions	AG-RDT without symptoms, PCR with symptoms			No restrictions	AG-RDT without symptoms, PCR with symptoms		
AG-RDT paired with PCRs	17,251	1,526	735	609	38,928	3,709	2,306	2,161
True positives	4,410	363	108	38	15,435	1,281	381	167
False positives	600	54	12	6	1,769	159	44	20
False negatives	1,078	199	190	218	3,531	856	1,007	1,182
True negatives	11,163	910	425	347	18,193	1,413	874	792

AG-RDTs antigen detection rapid diagnostic test; ISIN: Czech Information System of Infectious Diseases; SARS-CoV-2: severe acute respiratory syndrome coronavirus 2.

^a^ The data on 14-day country-level incidence were sourced from [25].

Table 2 presents the sensitivities for AG-RDTs conducted on the same day as the PCR. Considering the retrospective character of the data, PCR and AG-RDT on the same day is a proxy for the AG-RDT test conducted close to the peak of viral load. The overall sensitivity of AG-RDTs for Omicron was significantly higher (p < 0.05) than for the Delta variant (81.4% vs 80.4%). The lowest sensitivity for Delta was observed in the subgroup of vaccinated people with a reinfection (a previous infection recorded before the positive AG-RDT result). While the sensitivity for this subgroup was significantly higher (p < 0.001) in the Omicron dataset, this combination still had the lowest sensitivity among all four subgroups created by combining vaccination and reinfection status. There were statistically significant differences between Delta and Omicron among the tests done for diagnostic and preventive reasons (indication), however, the differences were small (less than 3%). Omitting the small outlying category of tests conducted for the reason ‘other’, we recorded the highest sensitivity for diagnostic tests and the lowest for preventive tests for both Delta and Omicron. Excluding the 7-day incidence levels of < 500 per 100,000, for which we did not have enough data for comparison, the sensitivity for all subgroups by incidence was consistently higher for Omicron than for Delta. In the age group ≥ 60 years, sensitivity for Delta was significantly lower (p < 0.001) than for Omicron. For both Omicron and Delta, sensitivity was the highest in the symptomatic subgroups. Among symptomatic persons, the percentage of cases manifesting individual symptoms remained similar, with the exception of the loss of smell and taste, which was less prevalent in the Omicron group. 

**Table 2:** Sensitivities of SARS-CoV-2 AG-RDTs done on the same day as the PCR (suspected higher viral load cases), Information System of Infectious Diseases, Czechia, December 2021–February 2022 (n = 5,488 PCR-positives for Delta and n = 18,966 PCR-positives for Omicron)

Subgroup (p value threshold^a^)	Delta			Omicron		
	Sensitivity (95% CI) in %	PCR-positive samples		Sensitivity (95% CI) in %	PCR-positive samples	
		n	%		n	%
Overall (p < 0.05)	80.4 (79.3–81.4)	5,488	100	81.4 (80.8–81.9)	18,966	100
Vaccination status						
Unvaccinated (p < 0.01)	80.0 (78.6–81.4)	3,096	56.4	82.0 (81.2–82.9)	8,798	46.4
Vaccinated	80.8 (79.2–82.4)	2,392	43.6	80.8 (80.0–81.6)	10,168	53.6
Previous COVID-19						
Not confirmed: All	80.9 (79.8–82.0)	5,295	96.5	81.9 (81.3–82.5)	15,306	80.7
Not confirmed: Vaccinated	81.5 (79.9–83.1)	2,333	42.5	81.5 (80.7–82.4)	8,406	44.3
Not confirmed: Not vaccinated (p < 0.05)	80.4 (79.0–81.9)	2,962	54.0	82.4 (81.5–83.3)	6,900	36.4
Confirmed: All (p < 0.001)	65.3 (58.1–72.5)	193	3.5	79.2 (77.8–80.5)	3,660	19.3
Confirmed: Vaccinated (p < 0.001)	52.5 (38.1–66.9)	59	1.1	77.4 (75.3–79.4)	1,762	9.3
Confirmed: Not vaccinated (p < 0.01)	70.9 (62.5–79.3)	134	2.4	80.8 (79.0–82.6)	1,898	10.0
Indication (reason for AG-RDT test)						
Diagnostic (p < 0.05)	83.4 (81.8–84.9)	2,463	44.9	85.1 (84.2–85.9)	6,790	35.8
Epidemiological	80.5 (77.6–83.3)	829	15.1	80.5 (79.3–81.7)	4,309	22.7
Preventive (p < 0.05)	73.0 (70.9–75.1)	1,834	33.4	75.5 (74.4–76.5)	6,720	35.4
Other	97.0 (94.9–99.0)	362	6.6	97.4 (96.4–98.4)	1,147	6.0
Regional SARS-CoV-2 incidence (new cases per 100,000 persons in 7 days before the AG-RDT)						
0–100	No data	0	0.0	No data	0	0.0
>100–≤500	74.4 (68.9–79.9)	277	5.1	62.5 (16.7–100)	8	0.0
>500–≤1,000 (p < 0.01)	81.2 (79.9–82.6)	3,404	62.0	83.7 (82.1–85.2)	2,259	11.9
>1,000–max	79.9 (78.0–81.8)	1,758	32.0	81.1 (80.5–81.7)	16,461	86.8
Region unknown	69.4 (54.5–84.3)	49	0.9	79.0 (73.4–84.6)	238	1.3
Age group (years)^b^						
0–12	81.9 (76.5–87.3)	232	4.2	79.8 (76.7–82.8)	722	3.8
13–18	77.8 (68.6–87.0)	99	1.8	80.5 (77.1–84.0)	560	3.0
19–25	83.4 (79.1–87.8)	320	5.8	80.5 (78.5–82.5)	1,585	8.4
26–59	82.5 (81.0–83.9)	2,804	51.1	81.3 (80.6–82.0)	11,482	60.5
≥ 60 (p < 0.001)	76.9 (75.0–78.8)	2,032	37.0	82.3 (81.2–83.4)	4,617	24.4
Presence of symptoms						
No symptoms reported (p < 0.01)	68.6 (66.1–71.1)	1,446	26.4	72.6 (71.4–73.8)	5,707	30.1
At least one symptom (p < 0.05)	83.8 (82.5–85.2)	3,068	55.9	85.2 (84.5–86.0)	9,735	51.3
Symptom data missing	86.9 (84.6–89.1)	974	17.8	85.0 (83.8–86.2)	3,524	18.6
Individual symptoms						
Cough (p < 0.05)	84.8 (83.3–86.4)	2,078	37.9	86.3 (85.4–87.2)	6,058	31.9
Muscle, joint pain, chills (p < 0.01)	89.3 (87.5–91.1)	1,252	22.8	86.0 (84.9–87.0)	4,443	23.4
Diarrhoea, vomiting	75.9 (68.8–83.0)	166	3.0	81.0 (76.6–85.3)	357	1.9
Temperature	87.4 (85.5–89.3)	1,279	23.3	85.9 (84.8–87.0)	3,934	20.7
Loss of smell, taste	84.5 (79.7–89.4)	252	4.6	88.5 (81.1–95.9)	96	0.5
Other symptoms (p < 0.01)	82.2 (79.7–84.8)	939	17.1	85.8 (84.6–87.0)	2,524	13.3
Selected less common test types						
Saliva	74.4 (59.1–89.7)	43	0.8	78.6 (71.9–85.3)	173	0.9
Fluorescence immunoassay	82.2 (72.1–92.3)	73	1.3	75.3 (68.1–82.6)	162	0.9
AG-RDT on EU Common List (16th update – July 2022) [5]						
EU Category A (n = 29; p < 0.01)^c^	79.3 (77.2–81.4)	1,571	28.6	82.8 (81.7–83.9)	4,693	24.7
EU Category B (n = 69)^c^	73.6 (70.0–77.2)	640	11.7	72.3 (70.5–74.1)	2,496	13.2
Not on Category A or B (n = 247; p < 0.05) ^c^	80.2 (78.5–81.9)	2,280	41.6	82.0 (81.2–82.8)	8,204	43.3
Test name not available or not resolved to EU test ID	86.8 (84.6–89.0)	997	18.2	84.5 (83.3–85.7)	3,573	18.8
AG-RDT on WHO EUL List (7 June 2022) [13]						
WHO EUL (n = 3; p < 0.05)^c^	79.9 (77.3–82.5)	960	17.5	83.0 (81.5–84.4)	2,648	14.0

AG-RDTs antigen detection rapid diagnostic test; CI: confidence interval; EU: European Union; EUL: Emergency Use Listing; ID: identification number; ISIN: Czech Information System of Infectious Diseases; SARS-CoV-2: severe acute respiratory syndrome coronavirus 2; WHO: World Health Organization.

^a^ p value threshold was included when the difference between Omicron and Delta was significant (p < 0.05).

^b^ There was one PCR-positive individual with a missing age record for the Delta subset.

^c^ The n indicates the count of distinct AG-RDT types included.

As the saliva and FIA tests accounted only for 0.8% and 1.3% of all PCR-positive results, we could compare the results of these tests with the overall average sensitivity reported in Table 2 (row *Overall*). Note that a comparison with a subgroup of all remaining tests would yield a nearly identical results due to the small share of saliva and FIA tests. For both Delta and Omicron, the sensitivity of saliva tests was lower than the average, but the difference was not significant. For Omicron, FIA tests had lower sensitivity compared with Delta, but the value was not statistically different from the average. From the three considered lists of approved AG-RDTs, the sensitivities of tests on the EU Category A list and the WHO EUL list were the highest and mutually similar (less than 1% difference).

Referring to the specificities for the same-day AG-RDT and PCR tests in Table 3, we observed a significantly (p < 0.001) lower specificity of AG-RDTs for Omicron compared with Delta. The specificity was lowest for the indication ‘other’, which we consider an outlier. The subgroup with loss of smell or taste had the second lowest specificity for Delta and the third lowest for Omicron, which indicates that people with these symptoms were more likely to receive a false positive AG-RDT result.

**Table 3:** Specificities of SARS-CoV-2 AG-RDTs done on the same day as the PCR (suspected higher viral load cases), Information System of Infectious Diseases, Czechia, December 2021–February 2022 (n = 11,763 PCR-negatives for Delta and n = 19,962 PCR-negatives for Omicron)

Subgroup (p value threshold^a^)	Delta			Omicron		
	Specificity (95% CI) in %	PCR-negative samples		Specificity (95% CI) in %	PCR-negative samples	
		n	%		n	%
Overall (p < 0.001)	94.9 (94.5–95.3)	11,763	100	91.1 (90.7–91.5)	19,962	100
Vaccination status						
Unvaccinated (p < 0.001)	92.6 (91.9–93.4)	4,510	38.3	87.5 (86.6–88.3)	6,233	31.2
Vaccinated (p < 0.001)	95.6 (93.7–97.6)	7,253	61.7	92.8 (92.4–93.3)	13,729	68.8
Previous COVID-19						
Not confirmed: All (p < 0.001)	94.9 (94.4–95.3)	9,502	80.8	91.5 (91.0–91.9)	14,829	74.3
Not confirmed: Vaccinated (p < 0.001)	96.2 (95.7–96.7)	5,818	49.5	93.2 (92.7–93.7)	10,299	51.6
Not confirmed: Not vaccinated (p < 0.001)	92.7 (91.8–93.5)	3,684	31.3	87.5 (86.5–88.5)	4,530	22.7
Confirmed: All (p < 0.001)	95.1 (94.2–96.0)	2,261	19.2	90.2 (89.4–91.0)	5,133	25.7
Confirmed: Vaccinated (p < 0.001)	96.6 (95.6–97.6)	1,435	12.2	91.7 (90.7–92.6)	3,430	17.2
Confirmed: Not vaccinated (p < 0.001)	92.5 (90.6–94.4)	826	7.0	87.3 (85.6–88.9)	1,703	8.5
Indication (reason for AG-RDT test)						
Diagnostic (p < 0.001)	93.3 (92.3–94.2)	2,818	24.0	89.8 (88.8–90.7)	4,374	21.9
Epidemiological (p < 0.001)	94.1 (93.1–95.1)	2,192	18.6	87.8 (86.8–88.8)	4,390	22.0
Preventive (p < 0.001)	96.2 (95.7–96.6)	6,693	56.9	93.7 (93.3–94.2)	11,050	55.4
Other (p < 0.01)	60.0 (46.0–74.0)	60	0.5	37.2 (28.7–45.6)	148	0.7
Regional SARS-CoV-2 incidence (new cases per 100,000 persons in 7 days before the AG-RDT)						
0–100	No data	0	0.0	No data	0	0.0
>100–≤500 (p < 0.05)	96.4 (95.1–97.6)	997	8.5	75.0 (8.1–100)	4	0.0
>500–≤1,000 (p < 0.001)	94.7 (94.2–95.2)	7,748	65.9	92.5 (91.4–93.5)	2,479	12.4
>1,000–max (p < 0.001)	94.8 (94.0–95.7)	2,764	23.5	91.0 (90.6–91.4)	17,165	86.0
Region unknown (p < 0.01)	94.9 (91.8–98.0)	254	2.2	88.5 (84.7–92.4)	314	1.6
Age group (years)						
0–12 (p < 0.01)	94.5 (92.8–96.3)	748	6.4	91.1 (89.2–93.1)	949	4.8
13–18 (p < 0.001)	95.6 (93.7–97.6)	504	4.3	88.7 (86.2–91.3)	657	3.3
19–25 (p < 0.001)	93.3 (91.5–95.0)	905	7.7	88.5 (86.7–90.2)	1,404	7.0
26–59 (p < 0.001)	94.0 (93.4–94.6)	5,819	49.5	89.0 (88.4–89.6)	10,532	52.8
≥ 60 (p < 0.01)	96.7 (96.1–97.3)	3,787	32.2	95.4 (94.9–95.9)	6,420	32.2
Presence of symptoms						
No symptoms (p < 0.001)	96.6 (96.1–97.1)	6,443	54.8	93.6 (93.1–94.0)	10,444	52.3
At least one symptom (p < 0.001)	93.2 (92.4–94.1)	3,551	30.2	87.6 (86.8–88.5)	5,958	29.8
Symptom data missing (p < 0.01)	92.0 (90.7–93.3)	1,769	15.0	89.9 (88.9–90.9)	3,560	17.8
Individual symptoms						
Cough (p < 0.001)	91.8 (90.6–93.1)	2,002	17.0	85.3 (84.0–86.6)	3,027	15.2
Muscle, joint pain, chills (p < 0.001)	88.5 (86.1–90.9)	772	6.6	79.4 (77.4–81.4)	1,691	8.5
Diarrhoea, vomiting (p < 0.001)	96.4 (93.8–98.9)	276	2.3	89.4 (86.4–92.5)	453	2.3
Temperature (p < 0.001)	91.5 (89.8–93.3)	1,040	8.8	83.7 (81.9–85.5)	1,710	8.6
Loss of smell, taste	76.5 (66.3–86.6)	85	0.7	80.7 (68.7–92.7)	57	0.3
Other symptoms (p < 0.001)	94.7 (93.5–95.9)	1,397	11.9	89.0 (87.8–90.3)	2,514	12.6
Selected less common test types						
Saliva	89.8 (82.8–96.8)	98	0.8	84.8 (77.7–91.9)	125	0.6
Fluorescence immunoassay	98.0 (96.2–99.7)	343	2.9	98.9 (97.9–100.0)	555	2.8
AG-RDT on EU Common List (16th update – July 2022) [5]						
EU Category A (n = 29; p < 0.001)^b^	96.9 (96.3–97.5)	3,831	32.6	94.6 (94.0–95.2)	5,792	29.0
EU Category B (n = 69; p > 0.01)^b^	94.4 (93.2–95.6)	1,561	13.3	92.6 (91.6–93.5)	2,917	14.6
Not on Category A or B (n = 247; p < 0.001)^b^	94.5 (93.8–95.2)	4,580	38.9	88.5 (87.8–89.2)	7,641	38.3
Test name not available or not resolved to EU test ID (p < 0.01)	92.0 (90.7–93.3)	1,791	15.2	90.0 (89.0–91.0)	3,612	18.1
AG-RDT on WHO List (7 June 2022) [13]						
WHO EUL (n = 3; p < 0.001)^b^	97.3 (96.6–98.0)	2,277	19.4	94.8 (94.0–95.5)	3,508	17.6

AG-RDTs antigen detection rapid diagnostic test; CI: confidence interval; EU: European Union; EUL: Emergency Use Listing; ID: identification number; ISIN: Czech Information System of Infectious Diseases; SARS-CoV-2: severe acute respiratory syndrome coronavirus 2; WHO: World Health Organization.

^a^ p value threshold was included when the difference between Omicron and Delta was significant (p < 0.05).

^b^ The n indicates the count of distinct AG-RDT types included.

Table 4 lists individual sensitivities for the AG-RDT types most commonly used in the Delta and Omicron periods. We used these data to evaluate to what extent the changes in the distribution of AG-RDT tests influenced the overall sensitivity. As detailed in the methodology section, we compared the average sensitivity of AG-RDTs in the Delta period with the average sensitivity recomputed to reflect the distribution of the tests in the Omicron period. Secondly, we compared the average sensitivity of AG-RDTs in the Omicron period with the sensitivity recomputed to reflect the distribution of the tests in the Delta period. Neither the difference between sens^Δ^ = 80.4% and sens^Δ^_o-distr_ = 80.7% nor the difference between sens^o^ = 81.4% and sens^o^_Δ-distr_ = 81.1% were statistically significant (p > 0.05).

**Table 4:** Sensitivities of SARS-CoV-2 AG-RDTs conducted on the same day as the PCR (suspected higher viral load cases), Information System of Infectious Diseases, Czechia, December 2021–February 2022 (total PCR-positive n = 5,488 for Delta and n = 18,966 for Omicron)

Test name	AG-RDT share of PCR-positives (%)		Sensitivity (%)	
	Delta	Omicron	Delta	Omicron
2019-nCoV Antigen Rapid Test Kit (Colloidal Gold Immunochromatography) - Beijing Lepu Medical Technology Co., Ltd	2.2	1.1	82.9	77.4
BIOSYNEX COVID-19 Ag BSS - BIOSYNEX S.A.	1.5	1.7	93.8	90.8
COVID-19 Antigen Detection Kit (Colloidal Gold) - Zhuhai Lituo Biotechnology Co., Ltd	1.8	1.2	46.4	53.3
COVID-19 Antigen Rapid Test Kit (Swab) - Safecare Biotech (Hangzhou) Co. Ltd	3.2	3.0	76.1	71.0
Flowflex SARS-CoV-2 Antigen Rapid Test - Acon Biotech (Hangzhou) Co., Ltd	1.6	1.7	85.6	90.0
Flowflex SARS-CoV-2 Antigen rapid test - ACON Laboratories, Inc (p < 0.001)	1.3	1.8	90.4	74.8
Humasis COVID-19 Ag Test - Humasis Co., Ltd.	8.5	7.7	79.2	79.4
NADAL COVID-19 Ag Test - Nal von minden GmbH	3.2	4.0	76.4	80.6
Panbio Covid-19 Ag Rapid Test - Abbott Rapid Diagnostics (p < 0.05)	15.2	11.8	79.1	82.3
SARS-CoV-2 Antigen Rapid Test Kit (Colloidal Gold Immunochromatography) - Beijing Lepu Medical Technology Co., Ltd	3.6	3.2	62.5	62.4
SARS-CoV-2 Antigen Test Kit - Shenzhen Ultra-Diagnostics Biotec.Co.,Ltd	1.1	1.5	95.2	91.6
SARS-CoV-2 Rapid Antigen Test - Roche (SD BIOSENSOR)	3.0	3.1	79.0	79.9
VivaDiag Pro SARS CoV 2 Ag Rapid Test - VivaChek Biotech (Hangzhou) Co., Ltd.	3.9	3.2	80.9	85.5
VivaDiag SARS CoV 2 Ag Rapid Test - VivaChek Biotech (Hangzhou) Co., Ltd	4.6	4.4	84.0	84.1
VivaDiag Wellion SARS-CoV-2 Antigen Rapid Test - VivaChek Biotech (Hangzhou) Co., Ltd.	1.6	2.3	83.3	86.9
Wellion SARS-CoV-2 PLUS ANTIGEN Rapid Test - MED TRUST Handelsges.m.b.H.	2.1	1.7	82.6	85.8
Wondfo 2019-nCoV Antigen Test (Lateral Flow Method) - Guangzhou Wondfo Biotech Co., Ltd (p < 0.05)	1.5	2.0	50.6	63.8
Less commonly used AG-RDTs (< 1% share)	19.6	23.6	81.1	82.5
Information on AG-RDT name not available	20.3	21.2	86.4	84.7
Weighted average			80.4	81.4

AG-RDTs antigen detection rapid diagnostic test; SARS-CoV-2: severe acute respiratory syndrome coronavirus 2.

Individually listed are all AG-RDTs with at least 1% share among the tests paired with a positive PCR for both Omicron and Delta. The reported percentages are rounded while the sum was computed on unrounded figures.

**Figure 2:**
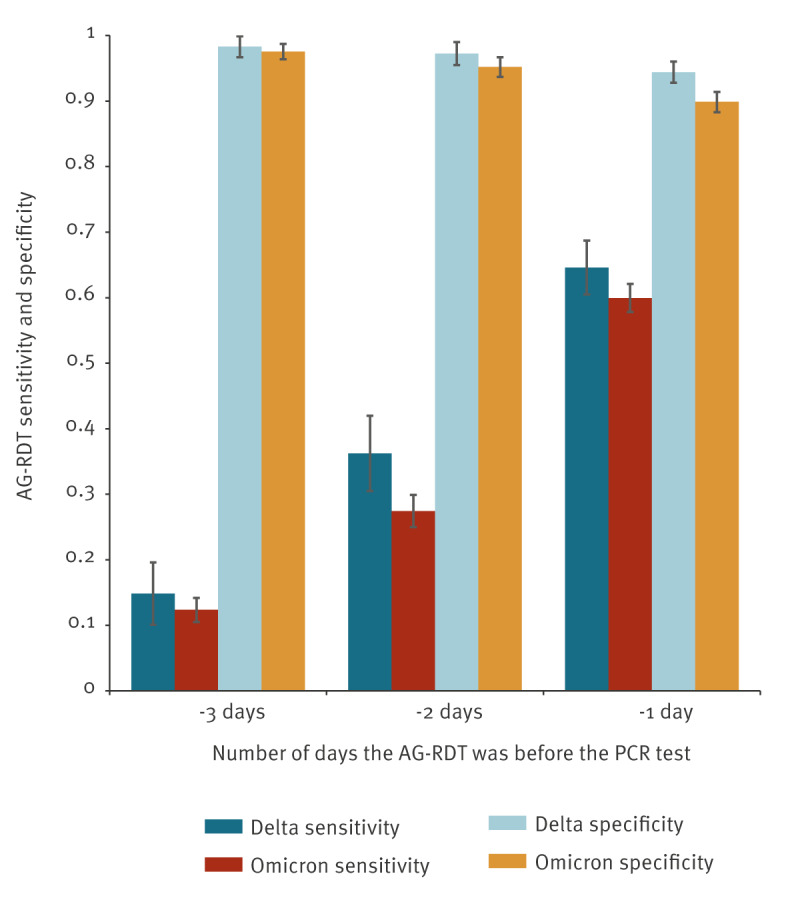
Sensitivity and specificity of AG-RDT tests done on asymptomatic individuals who reported symptoms at the time of a subsequent PCR test, Information System of Infectious Diseases, Czechia, December 2021–February 2022 (n = 2,870 for Delta and n = 8,176 for Omicron)

AG-RDT: antigen detection rapid diagnostic test; SARS-CoV-2: severe acute respiratory syndrome coronavirus 2.

To identify cases who took an antigen test at the beginning of the infection, we used a subset of cases where the person did not report symptoms at the time of the AG-RDT but did report symptoms at the time of the subsequent PCR. Figure 2 shows that the sensitivity of AG-RDT tests in this subset was consistently lower for Omicron than for Delta and that the earlier the AG-RDT was done, the lower the sensitivity.

This figure includes data obtained across all AG-RDT types. Shaded areas represent 95% confidence intervals.

The results for logistic regression are shown in Table 5. There was a strong positive and significant (p < 0.001) association between a true positive AG-RDT result and ‘other’ reason for the test (OR = 6.74 for Delta, 6.80 for Omicron), which was, however, infrequent (only 6.0 and 6.6% of PCR-positive cases according to Table 2). There was a negative and significant (p < 0.001) association between past COVID-19 infection and a true positive AG-RDT result for both Delta (OR = 0.53) and Omicron (OR = 0.83). With the exception of diarrhoea and vomiting, which were negatively but insignificantly associated, the presence of individual symptoms was positively associated with a true positive AG-RDT result for both Omicron and Delta. Focusing on Omicron, a significant association was observed for cough (OR = 1.51; p < 0.001), a symptom group described as ‘muscle, joint pain, chills’ (OR = 1.26; p < 0.001), temperature (OR = 1.19; p < 0.01) and ‘other symptoms’ (OR = 1.38; p < 0.001). For Delta, the age group ≥ 60 years was negatively associated (OR = 0.66; p < 0.05) with true positive AG-RDTs. There was no significant association between age and true positive AG-RDTs for Omicron. Vaccination was positively associated with a true positive result for Delta (OR = 1.17; p < 0.05) and negatively for Omicron, although the latter result was not statistically significant.

**Table 5:** Multivariable logistic regression of factors contributing to true positive SARS-CoV-2 AG-RDT result, based on results when AG-RDT and PCR tests were done on the same day, Information System of Infectious Diseases, Czechia, December 2021–February 2022 (n = 4,473 for Delta and n = 15,226 for Omicron)

Characteristics	Delta			Omicron		
	OR	95% CI	p value	OR	95% CI	p value
Previous COVID-19 infection	0.53	0.37–0.75	0.000	0.83	0.75–0.92	0.000
Vaccination	1.17	1–1.37	0.046	0.94	0.87–1.03	0.167
COVID-19 Incidence	1.00	1.00–1.00	0.186	1.00	1.00–1.00	0.001
Reason for AG-RDT test						
Preventive	Reference					
Diagnostic	0.88	0.69–1.12	0.296	0.94	0.84–1.06	0.348
Epidemiological	0.88	0.70–1.11	0.270	0.92	0.81–1.04	0.193
Other	6.74	3.43–13.25	0.000	6.80	4.60–10.04	0.000
Age (years)						
0–12	Reference					
13–18	0.76	0.39–1.47	0.415	1.02	0.72–1.45	0.901
19–25	1.07	0.64–1.78	0.806	0.92	0.71–1.20	0.546
26–59	1.00	0.67–1.50	1.000	1.08	0.85–1.36	0.527
≥ 60	0.66	0.43–0.99	0.047	1.05	0.82–1.33	0.723
Symptoms on AG-RDT test						
Cough	1.25	1.03–1.52	0.023	1.51	1.35–1.68	0.000
Muscle, joint pain, chills	1.95	1.57–2.42	0.000	1.26	1.13–1.41	0.000
Diarrhoea, vomiting	0.72	0.49–1.06	0.100	0.86	0.65–1.14	0.288
Temperature	1.58	1.28–1.94	0.000	1.19	1.06–1.34	0.003
Loss of smell, taste	1.02	0.71–1.47	0.914	1.34	0.70–2.54	0.373
Other symptoms	1.18	0.96–1.44	0.120	1.38	1.23–1.55	0.000

AG-RDTs antigen detection rapid diagnostic test; CI: confidence interval; OR: odds ratio; SARS-CoV-2: severe acute respiratory syndrome coronavirus 2.

Reference for previous COVID-19 infection was no record of a previous COVID-19 infection. Reference for vaccination was not being vaccinated. Reference category for binary variables (individual symptom categories) was not reporting any of these conditions. Records with missing values were omitted (1,015 for Delta and 3,740 for Omicron).

## Discussion

In the present study, the sensitivity of AG-RDTs for Omicron was 1% higher than for Delta (80.4% vs 81.4%), which is a small but statistically significant difference (p < 0.05). However, when the analysis was restricted to individuals who were asymptomatic at the time of antigen testing but reported symptoms when a subsequent positive PCR was done 1–3 days later, the sensitivity of AG-RDTs was lower for Omicron than for Delta. An explanation is that test results in individuals infected with the Omicron variant are positive for a shorter period of time than in those infected with the Delta variant [16]. In data collected in 2020, the risk of a false negative AG-RDT result was elevated when testing at an early stage of infection [17]. Our results based on field data show that antigen tests may be even less reliable at an early stage of an Omicron infection, supporting earlier analytical results of lower sensitivity for Omicron in asymptomatic individuals and during the early symptomatic period [18].

The effects of prior infection and vaccination on AG-RDT sensitivity have to our knowledge not been previously studied in large cohorts. The observation that vaccination was not associated with lower sensitivity for Delta concurs with the finding that peak viral load for the Delta variant did not differ by vaccination status [1]. The higher sensitivity for unvaccinated Omicron cases we observed is compatible with reports of higher viral load for mildly symptomatic Omicron infections in unvaccinated individuals [19]. We hypothesise that the lower sensitivity for individuals with Delta or Omicron infection who had a previously confirmed SARS-CoV-2 infection may be an effect of the immunity already primed by the prior infection, leading to lower viral load. This is supported by a report of lower viral load, for both Delta and Omicron, in reinfected individuals compared with unvaccinated individuals [19], and by data from the United Kingdom (UK) (April 2020 to 5 June 2021), where viral loads were typically lower in reinfection episodes compared with the initial infection [20]. It should be noted that research also indicates that the decrease in viral load due to vaccination diminishes with increasing time between the vaccination and reinfection [21] and that viral load, as measured by quantification cycle values, has a low to moderate correlation with concentrations of infectious virus particles for both the Omicron and Delta variants (both in vaccinated and unvaccinated people) [19]. These findings combined may potentially predict mean lower sensitivity of AG-RDTs in the future as the share of vaccinated and reinfected in the population and the share of preventive tests – where infected participants may have on average lower viral loads – will increase.

A recent study (n = 120 Omicron PCR-positive children ≥ 5 years) suggested that AG-RDT performance was preserved in children during the Omicron wave [22]. Our study supports this conclusion using statistical significance testing on a larger sample (n = 1,282 Omicron PCR-positive samples, age groups 0–12 and 13–18 years) compared with 331 Delta PCR-positives (same age groups).

The most distinct pattern specific to Omicron is that the loss of smell or taste is an almost 10 × less common symptom than it was for Delta, which has been confirmed by the marked reduction in this symptom for Omicron BA.1 infections in the UK [23].

In agreement with an early report relating to PCR tests [24], we observed a higher sensitivity of saliva tests for Omicron. This effect can be attributed to improved viral replication in upper respiratory tract tissue [24]. However, saliva test sensitivity was still below the average AG-RDT sensitivity.

The results did not show a significant difference in sensitivity between AG-RDTs on the EU Common List of Antigen Tests (Category A) and those on the reference WHO EUL, which was earlier deemed the best-performing list [6]. While the utility of the WHO EUL was limited for us as the version we used contained only eight AG-RDTs, the version of the EU Common List Category A covered a wider selection of 58 AG-RDTs in our study. The lower average sensitivity of AG-RDTs on the Category B list was in line with our expectation related to the less stringent demands on the evaluation of tests on this list.

We observed a lower average specificity of AG-RDTs for Omicron compared with Delta. We hypothesise that this could be partly related to a surge of infections, as the average incidence during the period when the Omicron dataset (3,814 new biweekly infections per 100,000) was collected was more than twice the average incidence registered in the period of collection of the Delta dataset (1,862 per 100,000). The incidence observed during the collection of the Omicron dataset was the highest ever recorded for SARS-CoV-2 in Czechia according to official daily incidence statistics published from 1 March 2020 to 5 May 2023 [25] when the WHO announced the end of the COVID-19 Public Health Emergency of International Concern [26]. A report about the testing network being overloaded during the time the Omicron dataset was collected also appeared in the Czech media [27].

Our study’s main limitations follow from its retrospective character. Information on viral load levels in tested individuals was not collected. Part of the individuals who were considered as having the first COVID-19 infection may actually account for reinfections, with the prior infection not being centrally registered, for example because the person was unaware of an infection with an asymptomatic course. The availability of time-stamped symptom data would allow us to more precisely determine at what stage of the infection the testing occurred. Due to the way the testing was organised in Czechia, the dataset with AG-RDT and PCR test results performed on the same day may be more representative of individuals with higher viral load. We have not evaluated to what extent the infected but AG-RDT-negative individuals were contagious. Analysing this would be a possible extension of our work as prior research has suggested that AG-RDT positivity better correlates with infectiousness than PCR positivity and thus could serve as an indicator for ending the isolation period [28].

## Conclusion

Our results indicate that when SARS-CoV-2 AG-RDTs are used in the early stage of infection, their sensitivity for infection with the Omicron variant is lower than with a Delta infection. Past SARS-CoV-2 infection was negatively associated with the sensitivity of AG-RDTs, it was the strongest predictor of a false negative AG-RDT result for both Delta and Omicron. For Omicron, we also observed a pattern of lower sensitivity in vaccinated individuals. The sensitivity in children was not lower compared with Delta, which suggests that in this age group, AG-RDTs are equally effective for Omicron. Our data support the choice of the EU Category A list over the B list. Further, our results suggest that AG-RDTs may not be suitable for the initial diagnosis as AG-RDTs may have low sensitivity in early asymptomatic phases of infection. AG-RDTs could, however, be a useful tool for checking the course of the infection and ending the isolation period as previously suggested. Large retrospective analyses of logs of paired AG-RDTs and RT-PCR results can lead to additional insights on the performance of AG-RDTs for specific subgroups, which can potentially lead to better diagnostic outcomes, improved availability of tests and reduced costs of public testing.
